# Deficiency of *NBAS* causes immune dysregulation due to hyperactivation of IFN signaling

**DOI:** 10.70962/jhi.20260018

**Published:** 2026-10-28

**Authors:** Chenlu Liu, Liang Zhang, Meiping Lu, Yunyan Shen, Yuhao Yao, Fei Liu, Qintao Wang, Benshan Zhang, Li Guo, Xiaozhong Li, Qing Zhou, Jianhua Mao, Xiaomin Yu

**Affiliations:** 1 State Key Laboratory of Common Mechanism Research for Major Diseases, Institute of Basic Medical Sciences & School of Basic Medicine, Chinese Academy of Medical Sciences & Peking Union Medical College, Beijing, China; 2 Department of Nephrology, Rheumatology and Immunology, The Affiliated Children’s Hospital of Xiangya School of Medicine, Central South University (Hunan Children’s Hospital), Changsha, China; 3 Department of Rheumatology, Immunology and Allergy, Children’s Hospital, Zhejiang University School of Medicine, Hangzhou, China; 4 Department of Nephrology and Immunology, Children’s Hospital of Soochow University, Suzhou, China; 5 Life Sciences Institute, Zhejiang University, Hangzhou, China; 6 Department of Nephrology, The Children’s Hospital, Zhejiang University School of Medicine, National Clinical Research Center for Child Health, Hangzhou, China; 7 Liangzhu Laboratory of Zhejiang University School of Medicine, Hangzhou, China; 8 Department of Hematology, The Affiliated Children’s Hospital of Xiangya School of Medicine, Central South University (Hunan Children’s Hospital), Changsha, China; 9 Department of Rheumatology, Sir Run Run Shaw Hospital, Zhejiang University School of Medicine, Hangzhou, China; 10 Department of Rheumatology, The Second Affiliated Hospital, Zhejiang University School of Medicine, Hangzhou, China

## Abstract

*NBAS* deficiency is a multisystem disorder, but its immunologic manifestations and underlying mechanisms remain poorly defined. Here, we expand the genotypic and phenotypic spectrum of *NBAS* deficiency through four patients carrying five loss-of-function *NBAS* mutations. Minigene assays and RNA analysis showed that these splicing or missense mutations resulted in aberrant alternative splicing. Clinically, the patients showed early-onset recurrent or persistent cytopenias and immune dysregulation, together with severe infections, inflammatory activation, and multi-organ involvement, including renal and neurologic manifestations. Transcriptomic analysis revealed overactivation of type I interferon signaling pathways compared with healthy controls, and single-cell RNA sequencing demonstrated that this upregulated inflammation was predominantly driven by myeloid cells. Together, these findings identify *NBAS* deficiency as a cause of immune dysregulation characterized by myeloid-driven type I interferonopathy, expand the clinical spectrum of *NBAS*-related disorders, and highlight the critical role of *NBAS* in maintaining immune homeostasis.

## Teaser

Liu et al. identify NBAS deficiency as an underrecognized cause of immune dysregulation. Patient transcriptomes reveal myeloid-driven type I interferon activation, linking impaired intracellular trafficking to interferonopathy and suggesting therapeutic potential for JAK inhibition.

## Introduction

Immune dysregulation diseases comprise a heterogeneous group of disorders characterized by aberrant immune responses and disrupted homeostasis. These conditions arise from defects in immune regulatory pathways, in which the genetic causative components play an important role in pathogenic mechanisms (1, 2).

Neuroblastoma amplified sequence (*NBAS*) encodes a subunit of the NRZ tethering complex, which is involved in Golgi-to-ER retrograde transport with the COPI complex. NBAS is a component of the trimeric NRZ complex together with RINT1 and ZW10, which interacts with ER SNAREs syntaxin 18, BNIP1, p31, and Sec22b and regulates the coupling and fusion of COPI with the ER. NBAS serves as a link between p31 and ZW10-RINT1 with its N-terminal and C-terminal region, respectively. NBAS depletion impairs COPI-dependent Golgi-to-ER retrograde transport of proteins (3, 4). Defects in retrograde transport from the Golgi back to the ER, which is essential for retrieving cycling and escaped resident ER proteins, have been described to cause human diseases. Coatomer subunit α (COPA) is a subunit of COPI complex, and its mutation causes autoimmune disease with elevated type I interferon (IFN-I) signaling (5, 6, 7, 8, 9). NBAS has also been reported to regulate nonsense-mediated decay (NMD) in human cells. Depletion of *NBAS* results in a significant increase in mRNA levels of NMD genes (10).

*NBAS* mutations have been reported to cause two major autosomal recessive disorders covering multiple systems, including short stature, optic nerve atrophy, and Pelger–Huët anomaly (SOPH) (OMIM #614800) (11) characterized by severe postnatal growth retardation, facial dysmorphism, short stature, and Pelger–Huët anomaly of leucocytes; and infantile liver failure syndrome 2 (ILFS2) (OMIM #616483) characterized by recurrent fever with acute liver failure (12). The development of hemophagocytic lymphohistiocytosis (HLH) has also been described as a clinical manifestation associated with *NBAS* mutations (13). Notably, ∼44% of patients with *NBAS* mutations exhibit immunologic impairment. However, previous investigations have been largely limited to characterizing quantitative and functional defects in specific lymphocyte subsets, such as natural killer (NK) and B cells (14, 15). The broader transcriptional landscapes and specific inflammatory signaling pathways across distinct cell lineages, particularly the contribution of myeloid cells to systemic inflammation, remain largely unexplored.

In this study, we report four patients with genetically confirmed *NBAS* deficiency who exhibited early-onset recurrent or persistent cytopenias, immune dysregulation, infection susceptibility, inflammatory activation, and multisystem involvement, including renal and neurologic manifestations. These findings expand the phenotypic spectrum of *NBAS*-related disease beyond its classical presentations. Using bulk RNA sequencing (RNA-seq) and single-cell RNA sequencing (scRNA-seq), we demonstrate for the first time that *NBAS* deficiency drives an elevation of IFN-I signaling, providing novel insights into the critical role of NBAS in maintaining immune homeostasis.

## Results

### Clinical information of patients

Four Chinese patients diagnosed with *NBAS* deficiency were included in this study (Table S1). Patient 1 (P1) was a male patient who initially presented with thrombocytopenia at 3 years of age, together with autoimmune features including positive antinuclear antibody (ANA) and anti-SSA antibody. His disease subsequently evolved into recurrent cytopenia and multisystem involvement. At ∼7 years of age, he developed his first ischemic stroke, followed by recurrent cerebral infarctions and progressive renal involvement with persistent proteinuria. Renal biopsy showed secondary membranous nephropathy-like changes with IgM deposition, and a later biopsy demonstrated membranoproliferative-like glomerular lesions considered related to immune complex and thrombotic microangiopathy-like injury. During the later disease course, he developed chronic kidney disease, severe recurrent infections, necrotizing oral/facial soft-tissue lesions with loss of palatal and gingival tissue, persistent anemia and thrombocytopenia, and marked lymphopenia. He received multiple immunomodulatory and supportive interventions, but these treatments resulted in only partial and transient improvement, and the disease remained relapsing and progressive. He later died from massive gastrointestinal bleeding.

Patient 2 (P2) was a male patient with early-onset renal and hematologic disease. He developed nephrotic syndrome–like manifestations in early childhood, with recurrent lower limb and eyelid edema and abnormal urinalysis. From ∼6 to 7 years of age, he had recurrent cytopenias involving platelets, hemoglobin, and leukocytes. The cytopenias were partially responsive to corticosteroids but repeatedly relapsed during tapering. Later hematologic evaluation documented severe thrombocytopenia and neutropenia. He subsequently developed nephrotic-range proteinuria, hypoalbuminemia, autoimmune hypothyroidism, and hypogammaglobulinemia. During a later admission, he developed severe pneumonia with respiratory failure, with respiratory syncytial virus positivity and molecular evidence of *Pneumocystis jirovecii* and cytomegalovirus infection. He received corticosteroids and other immunomodulatory therapies, together with supportive care. Edema and respiratory manifestations improved after treatment, and proteinuria decreased; however, the hematologic and renal disease followed a recurrent course of proteinuria.

Patient 3 (P3) was a 4-year-old girl who presented with thrombocytopenia for more than 1 mo, malnutrition, mild pallor, and hepatosplenomegaly. Initial laboratory testing showed anemia, thrombocytopenia, elevated inflammatory markers, liver dysfunction, hypokalemia, metabolic acidosis, and positive antinuclear antibody. During hospitalization, she fulfilled five HLH-2004 diagnostic criteria (16) and was clinically diagnosed with HLH, including recurrent fever >38.5°C, hepatosplenomegaly/splenomegaly, cytopenias affecting at least two lineages, bone marrow hemophagocytosis, and markedly elevated soluble IL-2 receptor/sCD25. Her cytopenias subsequently worsened, with severe thrombocytopenia and anemia, increased inflammatory cytokines, positive platelet antibody screening, and viral coinfection with cytomegalovirus and rhinovirus. She later deteriorated with difficult-to-control epistaxis, marked cytokine activation, and high proportions of activated CD8 T cells. After ruxolitinib-based treatment combined with corticosteroids and supportive care, bleeding and inflammatory activity improved, platelet counts recovered, C-reactive protein (CRP) normalized, and soluble IL-2 receptor decreased. After discharge, she underwent hematopoietic stem cell transplantation at another hospital and died from transplant-related complications.

Patient 4 (P4), the younger sister of P3, was a 2-year-old girl who presented with pallor for more than 6 mo. Unlike P3, she had a milder hematologic phenotype characterized mainly by anemia, leukopenia, and neutropenia, while platelet counts were preserved and no frank HLH crisis was documented. Bone marrow evaluation showed abnormal hematopoiesis with decreased granulopoiesis, active erythropoiesis, and no evidence of leukemia. She also had mild inflammatory and immune activation markers, including elevated soluble IL-2 receptor and CXCL9. She received anti-infective treatment, red blood cell transfusion, intravenous immunoglobulin, prednisone, and supportive care. Her anemia and general clinical condition improved, although neutropenia persisted.

Overall, all four patients showed early-onset and persistent or recurrent cytopenia as a prominent shared manifestation of *NBAS* deficiency. Patients in this study also exhibited immune dysregulation, including infection susceptibility, autoimmune features with positive autoantibodies and inflammatory activation, and multisystem involvement affecting the renal and neurologic systems. These clinical features, including antinuclear antibody (ANA)/anti-SSA positivity, immune complex–mediated glomerular disease, and recurrent childhood ischemic stroke, extended beyond the classical phenotypic spectrum of *NBAS* deficiency (Table 1).

**Table 1.** Comparison of clinical phenotypes across *NBAS* deficiency, including SOPH, ILFS2, HLH, and P1–P4 in this study

Feature	SOPH	ILFS2	HLH	P1	P2	P3	P4
Hematologic system	Pelger–Huët anomaly (hypolobulated granulocyte nuclei)	Coagulopathy during episodes; cytopenias may occur during severe episodes in some patients	Cytopenias, bone marrow hemophagocytosis	Early-onset thrombocytopenia from age 3 years; recurrent pancytopenia with anemia and thrombocytopenia	Recurrent trilineage cytopenia from 6 years old; severe thrombocytopenia during relapses	Thrombocytopenia at presentation, progression to severe cytopenia; marrow hemophagocytosis and marrow hypoplasia	Anemia with leukopenia and preserved platelets; bone marrow showed hypoplastic marrow
Immune system/infections	Recurrent infections, hypogammaglobulinemia and lymphopenia, and NK/B cell abnormalities have been reported in some patients	Acute infection or fever commonly triggers liver crises	Acute infections, recurrent fever, hyperferritinemia, and immune activation	Severe pneumonia and facial infection with multi-drug-resistant organisms; severe T, B, and NK cell lymphopenia; hypocomplementemia; ANA positivity (1:320) and anti-SSA positivity; elevated CRP	Severe pneumonia with respiratory failure; hypogammaglobulinemia with markedly reduced IgG and IgA	Infections; elevated CRP, elevated sCD25, activated CD8 T cell expansion, and abnormal CD107a degranulation; ANA positivity (1:320, homogeneous pattern); platelet antibody positivity	Bacterial respiratory infection; elevated ESR, sCD25, and CXCL9
Liver	Intermittent mild liver-function-test abnormalities in some patients	Febrile-triggered recurrent acute liver failure	Hepatomegaly and elevated transaminases may occur during HLH	​	Elevated ALT/AST during the disease course	Hepatosplenomegaly and elevated ALT/AST during active disease	Elevated AST
Kidney	​	​	​	Proteinuria since childhood; renal biopsies showing membranous nephropathy-like and membranoproliferative glomerulopathy-like lesions with immune complex injury; chronic kidney disease	Recurrent edema and nephrotic syndrome–like disease from early childhood; nephrotic-range proteinuria	Renal tubular acidosis, renal medullary echogenicity, and left renal pelvic separation	​
Growth/Skeletal system	Postnatal growth failure (short stature), micromelia, brachydactyly, wormian bones, osteopenia; fractures, cervical spine issues in some patients	​	​	​	​	​	​
Eye	Bilateral optic nerve atrophy with nonprogressive visual loss; achromatopsia, nystagmus, myopia in some patients	​	​	​	​	Ophthalmic evaluation showed thinning of the average macular ganglion cell layer/inner plexiform layer	​
Nervous system/development	Normal intellect; some have mild intellectual disability, developmental delay	Encephalopathy during episodes in some patients	​	Recurrent childhood ischemic strokes, with later recurrence and residual neurologic deficits	​	​	​
Metabolism/endocrine system	Ketotic hypoglycemia reported in some patients	Hypoglycemia and lactic acidosis during episodes in some patients	​	Metabolic acidosis and hyperlipidemia	Autoimmune thyroiditis with hypothyroidism and elevated anti-TPO and anti-thyroglobulin antibodies; hypoproteinemia	Hypokalemia, metabolic acidosis, and hyperinsulinemia	​
Skin/connective tissue	Loose, progeroid-appearing skin with decreased turgor; cutis laxa	​	​	Puffiness of both lower limbs	Recurrent eyelid and lower limb edema associated with nephrotic-range proteinuria	Malnutrition with nearly absent subcutaneous fat	Pale complexion, lack of subcutaneous fat
IFN signature	NA	NA	NA	Prominent IFN-I signature in PBMC RNA-seq data	NA	Prominent IFN-I signature in CD14 and CD16 monocytes in bone marrow scRNA-seq data	NA
Treatment	Supportive management, including treatment of infections and management of growth, skeletal, ophthalmologic, and immune complications when present	Supportive management including fever control, avoidance of catabolism, glucose support, hepatoprotective therapy	HLH-directed immunomodulatory therapy; hematopoietic stem cell transplantation may be considered in familial or refractory disease	Corticosteroids, multiple immunosuppressants, IVIG; partial and transient responses with subsequent relapses	Corticosteroids and immunosuppressive therapy, with IVIG for severe infection; improvement in proteinuria and respiratory status	Corticosteroids, ruxolitinib, IVIG; improvement in cytopenia, bleeding, inflammatory markers, liver dysfunction, and sCD25 levels	Antibiotics, IVIG, corticosteroids, transfusion; improvement in anemia and general condition, with persistent neutropenia

SOPH, short stature, optic nerve atrophy, and Pelger–Huët anomaly; ILFS2, infantile liver failure syndrome 2; HLH, hemophagocytic lymphohistiocytosis; IVIG, intravenous immunoglobulin; NA, not available; TPO, thyroid peroxidase.

### Patients with the *NBAS* variants

**Figure 1.**
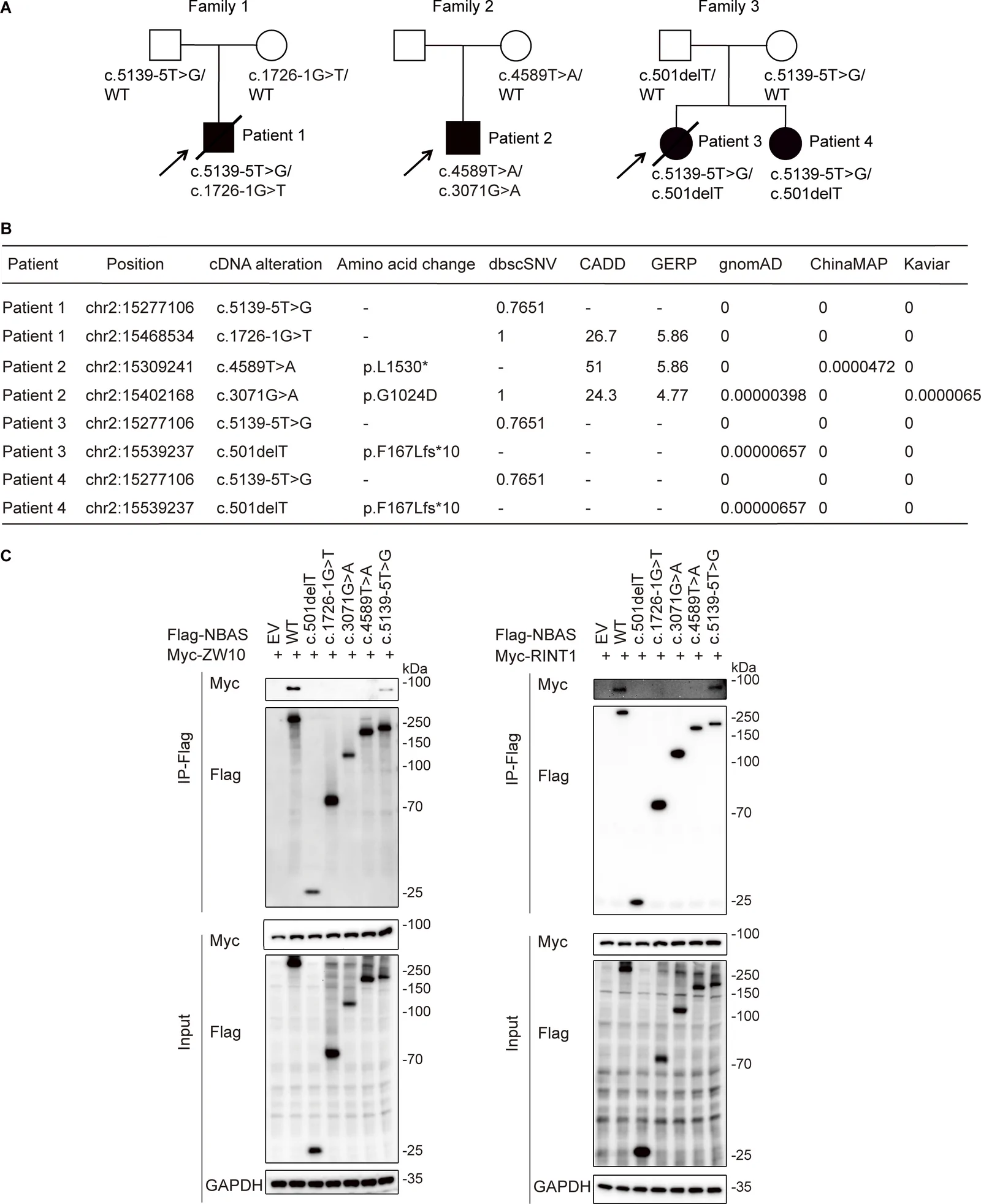
**Identification of patients with immune dysregulation carrying compound-heterozygous loss-of-function mutations in *NBAS*. (A)** Pedigrees showing compound-heterozygous *NBAS* mutations. Squares represent males, and circles represent females. Filled symbols indicate affected individuals, while open symbols represent unaffected family members. The proband is indicated by an arrow. Genotypes are shown below each individual. **(B)** In silico analysis of the compound-heterozygous *NBAS* mutations and the allele frequency in databases. The table lists the genomic positions (based on GRCh38), cDNA alterations, and corresponding amino acid changes for the identified mutations in patients. Pathogenicity and evolutionary conservation were evaluated using dbscSNV (for splicing variants), CADD, and Genomic Evolutionary Rate Profiling (GERP) scores. High dbscSNV scores (>0.6) suggest disruption of splicing. Minor allele frequencies are provided from gnomAD, ChinaMAP, and Kaviar databases. Hyphens (-) indicate splicing variants with no predicted amino acid substitution or scores not available in the databases. **(C)** Co-immunoprecipitation (IP) of FLAG-tagged wild-type (WT)/mutated NBAS and Myc-tagged ZW10 (left) or RINT1 (right) co-expressed in HEK293T cells as indicated. Immunocomplexes precipitated with anti-FLAG M2 Magnetic Beads were analyzed with antibodies as indicated. The image is representative of two independent experiments. EV, empty vector. Source data are available for this figure: SourceData F1.

**Figure S1.**
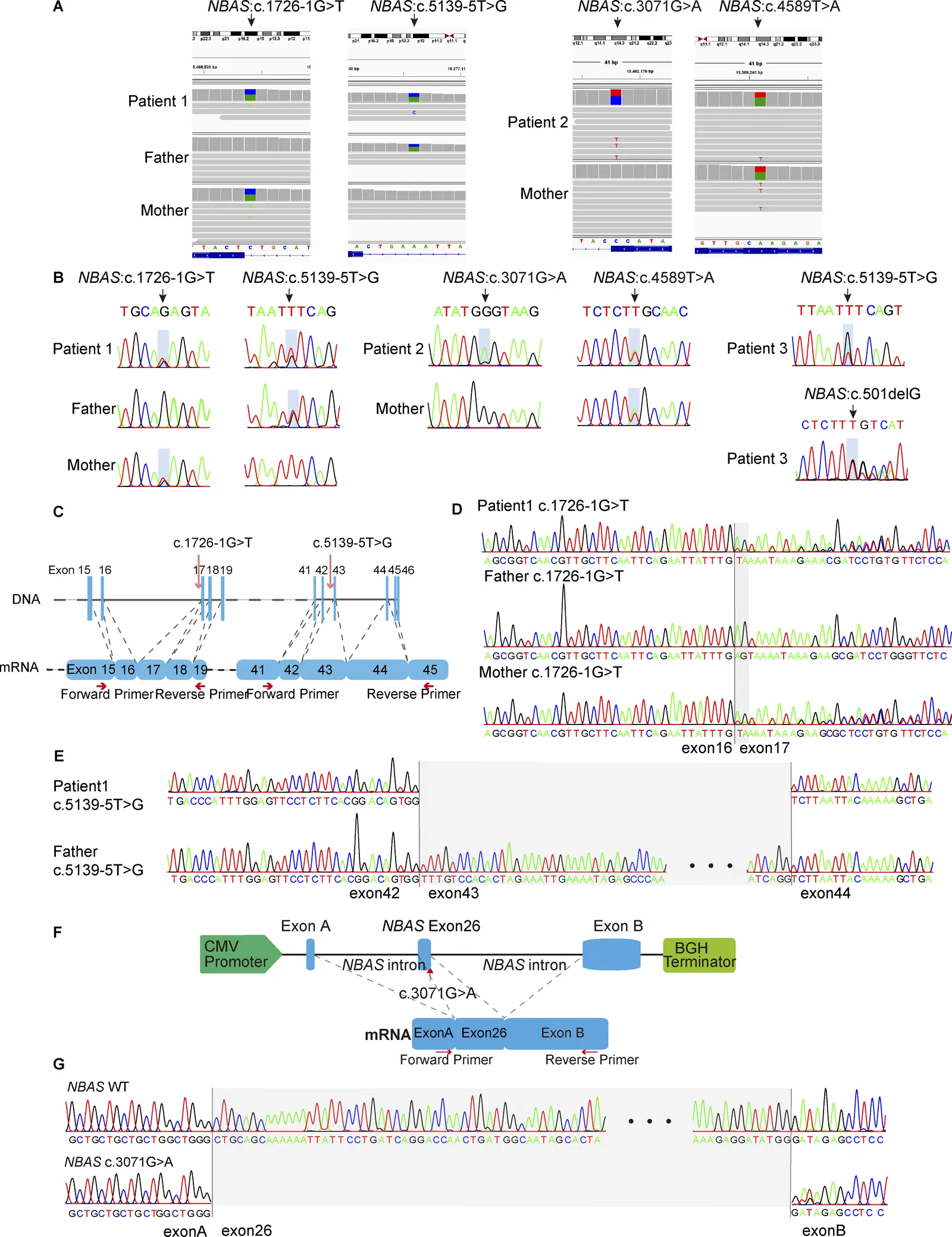
**Identification of loss-of-function mutations in *NBAS* in the patients. (A)** Integrative Genomics Viewer snapshots of exome sequencing reads showing the four identified variants in the probands. **(B)** Sanger sequencing verification of compound-heterozygous variants in *NBAS*. **(C)** RT-PCR analysis of *NBAS* mRNA transcripts from P1 and his parents. Primers were designed to amplify the regions spanning exons 15–19 and exons 41–45. **(D)** Sanger sequencing verification of aberrant alternative splicing on exon 17. **(E)** Sanger sequencing verification of aberrant alternative splicing on exon 43. **(F)** Schematic representation of the minigene reporter constructs used to characterize the splicing effect of the c.3071G>A mutation. The genomic region containing the mutation was cloned into a vector under the control of a CMV promoter. **(G)** In vitro splicing assay in HEK293T cells. RT-PCR amplification and Sanger sequencing of the minigene transcripts demonstrate that the c.3071G>A mutation leads to exon 26 skipping compared with the WT control. The image is representative of two independent experiments. BGH, bovine growth hormone; WT, wild type.

Whole-exome sequencing (WES) revealed the presence of five splicing or stop *NBAS* mutations in three families. P1 was compound-heterozygous for the c.5139-5T>G and c.1726-1G>T, and P2 was compound-heterozygous for the c.4589T>A (p.L1530*) and c.3071G>A (p.G1024D) *NBAS* (RefSeq: NM_015909.4) variants (Fig. 1 A and Fig. S1 A). P3 and P4 were compound-heterozygous for the c.5139-5T>G and c.501delT (p.F167Lfs*10) (Fig. 1 A). Among these mutations, only c.5139-5T>G and c.501delT have been previously reported (17, 18), while other mutations were novel. These mutations were rare and not present as homozygotes in public databases, including gnomAD, ChinaMAP, and Kaviar. In addition, these mutations were predicted to be deleterious by multiple software. The stop mutation c.4589T>A (p.L1530*) was a predicted loss-of-function variant with combined annotation-dependent depletion (CADD) Phred score of 51, and the splicing or missense mutations were predicted to be pathogenic with the CADD Phred score of 26.7 and 24.3, respectively (Fig. 1 B). Sanger sequencing validated the identified mutations and confirmed their compound-heterozygous state (Fig. S1 B). These data suggested that the clinical phenotypes in the three families were inherited recessively and segregated with the inheritance of *NBAS*, implying the potential pathogenic role of these *NBAS* variants.

### *NBAS* splicing or missense mutations cause aberrant alternative splicing

As the mutations c.1726-1G>T and c.5139-5T>G were located in the intron of *NBAS* and the mutation c.3071G>A was located in the last nucleotide of the exon, these mutations were presumed to cause aberrant alternative splicing. In addition, these mutations were predicted to be splice altering variants with the dbscSNV value of 0.7651 or 1 (Fig. 1 B). To confirm the predicted impact of these mutations on *NBAS* gene splicing, we extracted and analyzed RNA from P1 and his parents. Sanger sequencing of the reverse transcription–PCR (RT-PCR) products showed that the mutation c.1726-1G>T caused the loss of the first two nucleotides of exon 17, introducing a premature stop codon (p.S576*), while the mutation c.5139-5T>G caused the deletion of exon 43 and the introduction of a premature stop codon (p.L1714Sfs*2) (Fig. S1, C–E). To verify the effect of mutation c.3071G>A, wild-type and mutant c.3071G>A genomic fragments of *NBAS* were cloned into the pCAS2 vector and HEK293T cells were transfected with the minigene. The mRNA sequence showed that the mutation c.3071G>A resulted in the deletion of exon 26 and introduction of a premature stop codon (p.L980*) (Fig. S1, F and G). As the C-terminal (amino acids 1,036–2,371) of NBAS is responsible for the interaction with ZW10-RINT1, and the truncated C-terminal NBAS has no interaction with ZW10 and RINT1 (3), we performed co-immunoprecipitation assays to directly evaluate whether these variants affect the interaction between NBAS and its binding partners. c.1726-1G>T, c.4589T>A, c.3071G>A, and c.501delT mutant NBAS proteins exhibited complete loss of interaction with ZW10 and RINT1, and c.5139-5T>G mutant NBAS proteins exhibited impaired interaction with ZW10 and RINT1, whereas the wild-type protein retained robust binding (Fig. 1 C). These results provide direct experimental evidence that the variants disrupt the formation of the NBAS–ZW10–RINT1 complex, supporting a loss-of-function mechanism.

### Elevated inflammatory signals in patient’s peripheral blood mononuclear cells (PBMCs)

Considering the essential role of NBAS in COPI-dependent retrograde transport, it is likely that *NBAS* deficiency mimics the pathogenic mechanism of COPI dysfunction, potentially leading to the overactivation of the IFN-I pathway.

**Figure 2.**
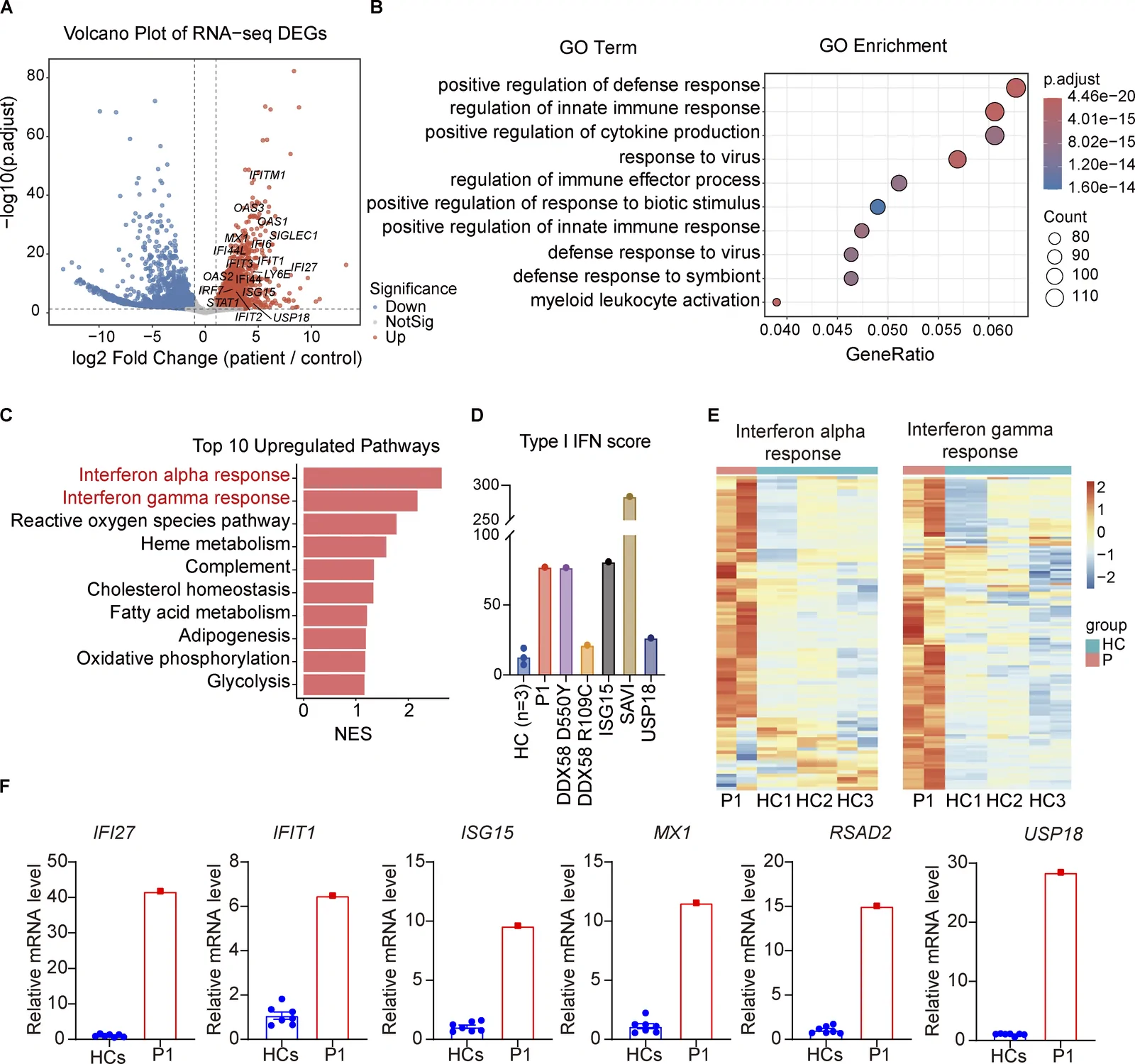
**Upregulated inflammation in patient’s PBMCs. (A)** Volcano plot displaying DEGs in PBMCs from P1 compared with HCs (*n* = 3). Red dots represent significantly upregulated genes (log_2_ fold change >1.0 and P < 0.05), and blue dots represent downregulated genes. Key ISGs are highlighted. **(B)** GO enrichment analysis of the upregulated DEGs. The top 10 significantly enriched biological processes are shown. **(C)** GSEA showing significant enrichment of IFN-α and IFN-ɣ pathways in P1 compared with HCs. **(D)** IFN-I score of HC (*n* = 3), P1, and positive disease controls with known type I interferonopathies determined by RNA-seq. Each dot represents one individual sample; duplicate libraries, where available, were combined before IFN score calculation. Note that the patient exhibits high IFN scores comparable to established interferonopathies. **(E)** Transcription level of the IFN-α pathway and IFN-ɣ pathway in PBMCs from P1 and HCs, determined by RNA-seq. Analysis of each sample was performed in duplicate. The genes included in each pathway are listed in Table S3. **(F)** mRNA expression levels of *IFI27*, *IFIT1*, *ISG15*, *MX1*, *RSAD2*, and *USP18* in PBMCs from seven HCs (*n* = 7) and P1 (*n* = 1) were quantified by qPCR. Bars represent the mean ± SEM. The qPCR assay was performed with technical triplicates for each sample. For panels A–E, bulk RNA-seq was performed using two independently prepared PBMC libraries. DEGs, differentially expressed genes; HCs, healthy controls; GO, Gene Ontology.

To verify whether *NBAS* deficiency is associated with the dysregulation of IFN-I production, we performed RNA-seq to profile the inflammatory transcriptome of P1 during persistent renal dysfunction and cytopenia. Immune-related genes including *IFI44L*, *OAS1*, *OAS3*, and *SIGLEC1* were upregulated in the patient, and differentially expressed genes in the patient compared with healthy controls were enriched in the immune response–regulating signaling pathway and the regulation of innate immune pathway (Fig. 2, A and B). Gene set enrichment analysis (GSEA) enrichment showed the elevation of interferon alpha (IFN-α) and interferon gamma (IFN-ɣ) pathways in P1 (Fig. 2 C). Since the IFN-I score is considered as an indicator of autoimmune and autoinflammatory disorders, we compared the IFN-I score between healthy controls, P1, and reported cases of monogenic interferonopathies including *DDX58* gain-of-function mutation (19, 20), *ISG15* deficiency (21), *STING* gain-of-function mutation (22), and *USP18* deficiency (23). The IFN-I score was markedly increased in P1 and fell within the range observed in patients with *DDX58* mutations and *ISG15* deficiency (Fig. 2 D). Heatmap analysis further confirmed that the transcriptional levels of several genes in IFN-α and IFN-ɣ pathways were elevated in P1 (Fig. 2 E). To validate these transcriptomic findings with laboratory tests, we performed quantitative real-time PCR (qPCR) on the PBMCs of P1. Consistent with the RNA-seq data, the qPCR results demonstrated a marked upregulation of interferon (IFN)-stimulated genes (ISGs), including *IFI27*, *IFIT1*, *ISG15*, *MX1*, *RSAD2*, and *USP18* (Fig. 2 F). Together, these results indicated that inflammatory responses, especially IFN responses, were elevated in the *NBAS*-deficient patient.

### Activated inflammation in patient’s bone marrow hematopoietic cells

**Figure 3.**
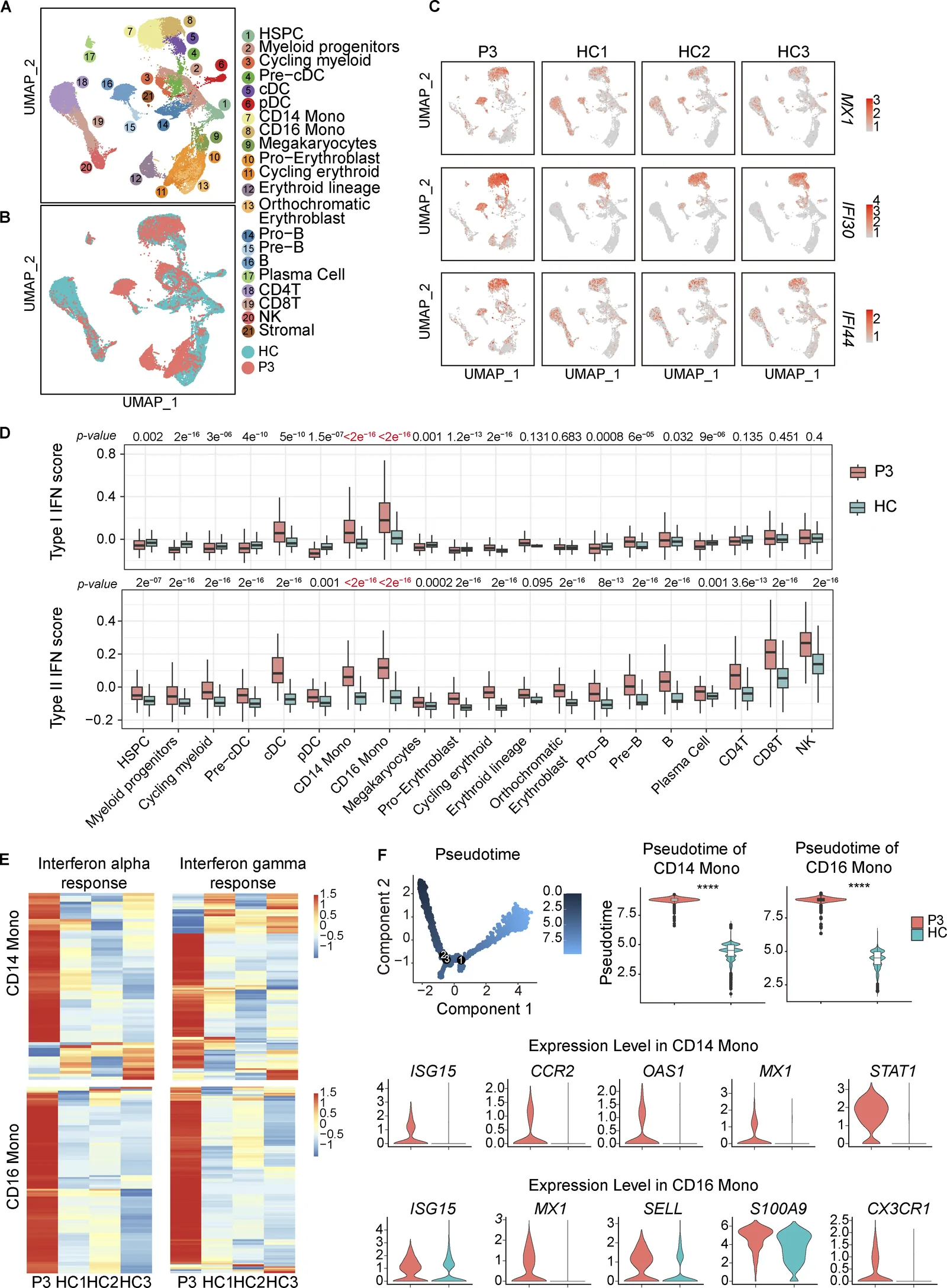
**Activated inflammatory signature in patient’s bone marrow hematopoietic cells. (A)** UMAP visualization of bone marrow hematopoietic cells from three healthy controls (HC1, *n* = 13,471 cells; HC2, *n* = 9,184 cells; HC3, *n* = 9,998 cells) and P3 (*n* = 7,370 cells) and marker-based annotation of 21 cell types. HSPC, hematopoietic stem and progenitor cell; CD14 Mono, CD14 monocyte; CD16 Mono, CD16 monocyte; conventional dendritic cell (cDC) and plasmacytoid dendritic cell (pDC). **(B)** UMAP visualization based on the sample type. Red dots represent patient, and blue dots represent healthy controls. **(C)** Single-cell expression profiles of key IFN-I–response genes (*MX1*, *IFI44*, *IFI30*) projected onto UMAP embeddings. Cells from healthy controls (HC1–3) and P3 are shown. The color gradient indicates relative gene expression levels. Note the generalized upregulation of these ISGs in the patient’s cells compared with controls. **(D)** IFN-I and type II IFN score in indicated cell types. Module scores were calculated for each cell based on canonical gene signatures. Box plots display the median (center line), interquartile range (IQR) (box limits), and whiskers extending to 1.5× IQR. Statistical significance between patient (red) and healthy controls (blue) within each cell type was assessed using the two-sided Wilcoxon rank-sum test (P values as indicated). Note the marked upregulation of IFN scores in patient, particularly in the myeloid compartment. **(E)** Transcription level of IFN-α response pathway and IFN-ɣ pathway response in CD14 monocytes and CD16 monocytes from P3 and healthy controls. The genes included in each pathway are listed in Table S3. **(F)** Pseudotime analysis of HSPC, myeloid progenitors, cycling myeloid, CD14 monocytes, and CD16 monocytes and expression level of inflammatory signatures and pseudotime in CD14 monocytes and CD16 monocytes from P3 and healthy controls. Statistical significance was assessed using the Wilcoxon rank-sum test. ****P ≤ 0.0001. Expression level of *ISG15*, *CCR2*, *OAS1*, *MX1*, and *STAT1* in CD14 monocytes and expression level of *ISG15*, *MX1*, *SELL*, *S100A9*, and *CX3CR1* in CD16 monocytes from healthy controls and P3. HSPC, hematopoietic stem and progenitor cell; UMAP, Uniform Manifold Approximation and Projection.

**Figure S2.**
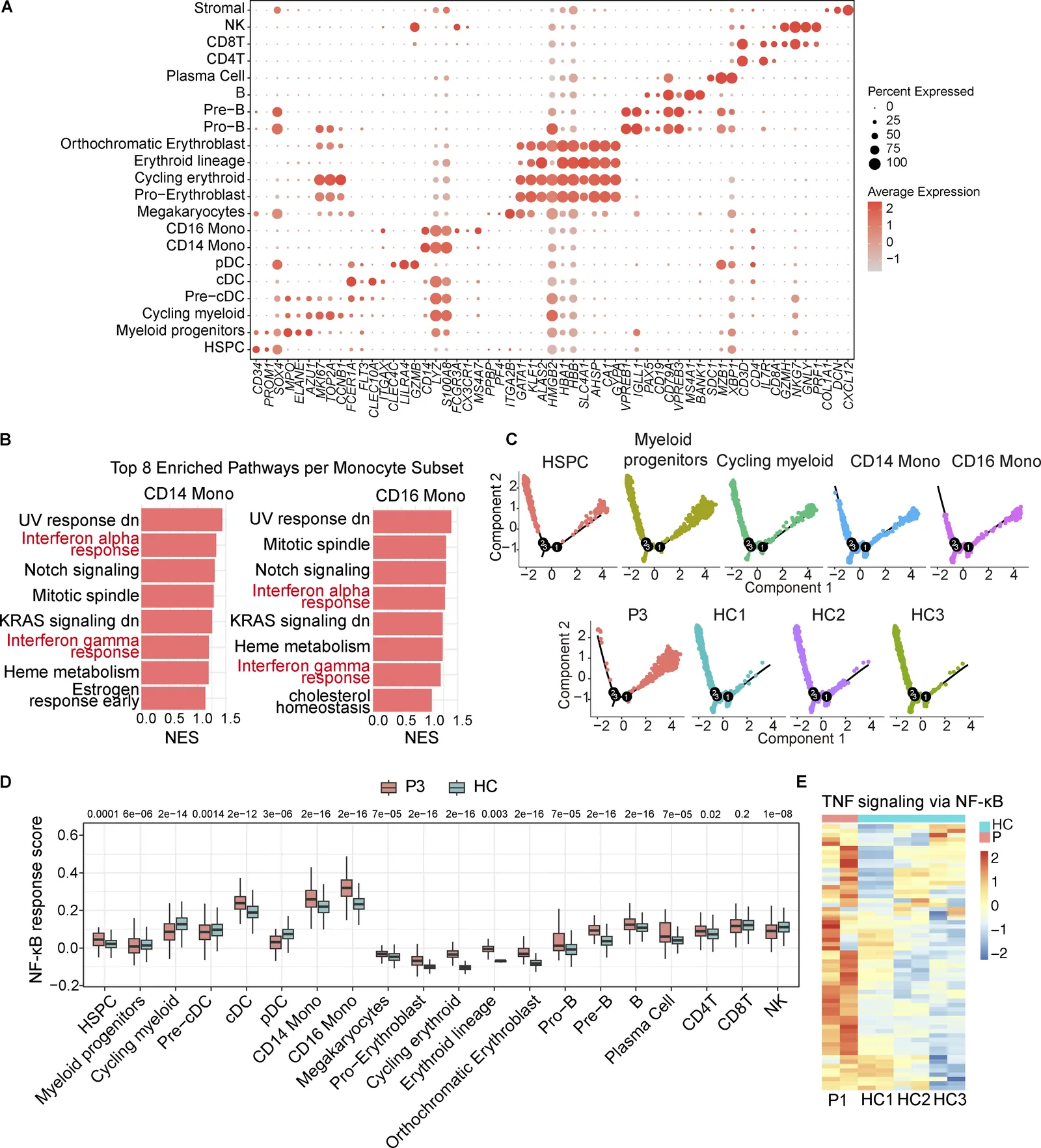
**Activated inflammation in patient’s bone marrow hematopoietic cells. (A)** Dotplot displaying the expression of canonical marker genes used for cell annotation. **(B)** GSEA showing significant enrichment of the IFN-α pathway and IFN-ɣ pathway in CD14 and CD16 monocytes of the patient compared with healthy controls (HCs), down (dn), and normalized enrichment score (NES). **(C)** Single-cell trajectory analysis (pseudotime) projected onto UMAP embeddings. Cells are colored by cell-type annotation (top) and sample origin (bottom), illustrating the differentiation continuum. **(D)** NF-κB response scores across indicated cell subsets in the P3. Data are presented as violin plots conventional dendritic cell (cDC) and plasmacytoid dendritic cell (pDC). **(E)** Heatmap showing the transcriptional levels of key genes involved in the NF-κB pathway in P1. The genes included in the pathway are listed in Table S3. HSPC, hematopoietic stem and progenitor cell; UMAP, Uniform Manifold Approximation and Projection.

To elucidate the impact of *NBAS* deficiency on immune profile, we performed scRNA-seq on bone marrow hematopoietic cells isolated from P3 during transient partial hematologic improvement before a subsequent HLH inflammatory flare. Dimensionality reduction and clustering analysis identified 29 distinct clusters, which were annotated based on canonical marker gene expression (Fig. 3, A and B; and Fig. S2 A). Proinflammatory mediators and ISGs including *MX1*, *IFI44*, and *IFI30* were transcriptionally upregulated in P3, especially in monocytes (Fig. 3 C). Analysis of IFN-I and type II IFN score confirmed the hyperinflammatory state in P3, particularly in myeloid cells (Fig. 3 D). GSEA and heatmaps revealed that gene sets, such as IFN-α pathway and IFN-ɣ pathway, were enriched in patient’s myeloid cells compared with healthy controls (Fig. S2 B and Fig. 3 E). Pseudotime trajectory analysis of hematopoietic stem and progenitor cells and myeloid subsets showed that CD14 and CD16 monocytes in the patient occupied a more advanced differentiation state and were more activated than healthy controls, confirmed by higher expression of inflammatory signature (Fig. 3 F and Fig. S2 C). Furthermore, NF-κB activation was also consistently observed in both P1 and P3 (Fig. S2, D and E). Collectively, these results demonstrated that *NBAS* deficiency drove a profound inflammatory response in bone marrow hematopoietic cells, which was predominantly sustained by the myeloid compartment.

## Discussion

In this study, we report four patients carrying compound-heterozygous *NBAS* variants. These patients exhibited an underrecognized immune dysregulation phenotype characterized by early-onset persistent or recurrent cytopenia, systemic inflammatory activation, recurrent infections, autoimmune features, and renal and neurologic involvement. Through comprehensive transcriptomic analysis of PBMCs and scRNA-seq of bone marrow hematopoietic cells, we demonstrate for the first time that *NBAS* deficiency drives a profound elevation of IFN-I signaling. This finding is clinically corroborated by the therapeutic efficacy of ruxolitinib, a JAK inhibitor targeting this pathway, in P3. These findings not only expand the genotypic spectrum of *NBAS* variants but also define a novel immune dysregulation phenotype driven by myeloid hyperactivation.

*NBAS* deficiency has increasingly been recognized as an inborn error of immunity. Our patients shared previously recognized immunologic features of *NBAS* deficiency, including recurrent infections, hypogammaglobulinemia, and B and NK cell abnormalities. Beyond these established features, patients in our study exhibited an expanded phenotype characterized by autoimmunity with ANA and anti-SSA positivity and immune complex–mediated glomerular disease. These manifestations overlap with the clinical spectrum of type I interferonopathies (24) and may be related to the prominent monocyte IFN activation identified in our study. Thus, monocyte IFN activation might represent an additional inflammatory mechanism contributing to the expanded immune phenotype of *NBAS* deficiency.

Mechanistically, it is likely that this IFN signature in our patients may arise from a defect in retrograde transport, similar to that observed in COPA syndrome, in which defective COPI-mediated STING retrieval causes constitutive IFN-I activation (5, 7, 9, 25). As NBAS cooperates with COPI-dependent retrograde transport as part of the NRZ tethering complex, *NBAS* deficiency may similarly disturb trafficking homeostasis and promote IFN activation. *DDX58*-, *ISG15*-, and *USP18*-associated interferonopathies converge on IFN-I activation through different mechanisms, including enhanced RIG-I–mediated nucleic acid sensing (19, 20), impaired ISG15-dependent USP18 stabilization (21), or defective negative feedback downstream of IFNAR signaling (23). In contrast, *NBAS* deficiency may link impaired intracellular trafficking to monocyte-driven IFN activation, suggesting a trafficking-associated route to interferonopathy-like immune dysregulation.

Our findings have potential clinical implications, particularly for *NBAS*-deficient patients with prominent inflammatory activation. The identification of a prominent IFN-I signature suggests that JAK inhibitors, which block IFN signaling, may offer a targeted therapeutic approach for this specific subset of *NBAS*-deficient patients. The clinical improvement observed in P3 following treatment with ruxolitinib combined with corticosteroids supports the potential efficacy of JAK inhibitors.

Several limitations of this study should be acknowledged. First, because *NBAS* deficiency is rare, the study size was small; therefore, the expanded immune phenotype described here will require validation in additional patients. Second, transcriptomic analyses support IFN-I activation as a feature of *NBAS* deficiency, longitudinal samples across active disease and remission will be needed to determine the persistence and dynamics of this IFN signature. Finally, the therapeutic response to ruxolitinib is based on a single patient. Future clinical evaluation of JAK inhibition in more *NBAS*-deficient patients will be essential to establish the efficacy and safety of this treatment approach.

In conclusion, we identify five *NBAS* mutations in four patients with a previously underrecognized immune dysregulation phenotype characterized by autoimmune features, inflammatory activation, and multisystem involvement, including renal, neurologic, and hematologic manifestations, expanding the phenotypic spectrum of *NBAS* deficiency. RNA-seq and scRNA-seq reveal the elevation of IFN-I pathways in the patients with *NBAS* deficiency. Inhibition of IFN activation could be a potential treatment for *NBAS*-deficient patients.

## Materials and methods

### Patients

The study protocol involving the patients was reviewed and approved by the Institutional Review Boards at Children’s Hospital, Zhejiang University School of Medicine (2021-IRB-172 and 2026-IRB-0094-P-01). Written informed consent was obtained from the adult participant and from the parents or legal guardians of minor participants, where applicable.

### WES and Sanger sequencing

Peripheral blood DNA was extracted using Maxwell RSC Whole Blood DNA Kit (AS1520; Promega). WES was performed using 1 μg of DNA. WES data analysis was conducted as previously described (26). The variants were annotated with ANNOVAR (2019Oct24) and filtered based on the frequency in gnomAD, Kaviar, and dbSNP databases. The variants identified by WES were validated by Sanger sequencing.

### qPCR

Total RNA was extracted from PBMCs with an RNeasy Mini kit (74104; Qiagen). The extracted RNA was first reverse-transcribed into cDNA and subsequently analyzed for qPCR.

### RNA-seq

Total RNA was isolated from PBMCs, and sequencing libraries were prepared using NEBNext Ultra RNA Library Prep Kit for Illumina (New England Biolabs). Libraries were subsequently sequenced on an Illumina NovaSeq platform to generate 150-bp paired-end reads. The reads were aligned to the human reference genome (GRCh38) using HISAT2, and gene-level read counts were obtained with featureCounts. Differential expression analysis was performed using the DESeq2 R package. The IFN score was calculated based on 28 IFN-I–response genes, as previously described (27). Data for other monogenic interferonopathies were obtained from cases previously reported by our group (18, 19, 20, 21, 22), and detailed genetic information is provided in Table S2.

### scRNA-seq

scRNA-seq was performed on bone marrow hematopoietic cells from the patient using the 10x Genomics Chromium platform. ScRNA-seq datasets of bone marrow hematopoietic cells from healthy controls were obtained from the Human Cell Atlas database (28, 29). Raw sequencing data were processed with Cell Ranger (v9.0.1), followed by downstream analysis using Seurat (v5.3.0). Pseudotime trajectory analysis was conducted with Monocle (v2.22.0) (30).

### Minigene assay

Minigene assay was used to investigate the mutation’s effect on RNA alternative splicing. Wild-type and mutant genomic fragments of *NBAS* comprising appropriate exons and introns were amplified with primers. The genome fragments were cloned into the pCAS2 vector and then expressed under the control of the CMV promoter in HEK293T cells. RNA was extracted 24 h after transfection, and then RT-PCR was performed.

### Co-immunoprecipitation and western blot

For co-immunoprecipitation, HEK293T cells were collected and incubated for 15 min in lysis buffer containing 0.5% NP-40. After centrifugation at 15,000 rpm for 15 min at 4°C, the soluble fractions were collected, and total protein concentrations were determined using a bicinchoninic acid protein assay kit (23225; Thermo Fisher Scientific). Cell lysates were subsequently incubated with anti-FLAG immunomagnetic beads (M8823; Sigma-Aldrich) overnight at 4°C. The bead-bound protein complexes were washed three times with lysis buffer and denatured for subsequent immunoblotting. Proteins were separated by sodium dodecyl sulfate–polyacrylamide gel electrophoresis and transferred to membranes using a wet-transfer system. After blocking with 5% nonfat milk, the membranes were incubated at 4°C overnight with primary antibodies against FLAG (rabbit monoclonal; #14793S; CST) or GAPDH (rabbit polyclonal; #ET1601-4; HUABIO). Appropriate horseradish peroxidase (HRP)–conjugated secondary antibodies were then applied. Myc-tagged proteins were detected directly using an HRP-conjugated anti-Myc antibody ( #2040S; CST). Immunoreactive bands were developed by enhanced chemiluminescence and recorded on film.

### Statistical methods

Single-cell gene signature scores were computed using the Seurat AddModuleScore function. Statistical comparisons between groups were performed using the two-sided Wilcoxon rank-sum test. A P value <0.05 was considered statistically significant.

### Reagents

Cell Lines: For cell culture, HEK293T cells were verified negative for *Mycoplasma* contamination.

Oligonucleotides: All primers were synthesized by Tsingke Biotechnology Co., Ltd. The specific primer sequences used in this study are listed as follows:

Primers for minigene plasmid construction:

Forward: 5′-CTG​ATA​CAT​AGC​TTA​TGG​GGT​TAC-3′

Reverse: 5′-TAT​AGG​CAA​AGG​CCA​AGA​AGA​AA-3′.

Primers for RT-PCR amplification:

Forward: 5′-GAA​TTC​GTC​CGC​TGA​CGT​CG-3′

Reverse: 5′-CTG​AGA​AGG​CGT​GGT​AGA​GC-3′.

Primers for amplifying the region spanning exons 15–19:

Forward: 5′-GGA​AAC​GCC​CAC​GAA​CCA​TT-3′

Reverse: 5′-CTT​CCG​TCT​ACA​ACG​GCA​AAG-3′.

Primers for amplifying the region spanning exons 41–45:

Forward: 5′-GGA​CGT​GCA​GCG​GTT​TAC​TG-3′

Reverse: 5′-CTC​AGG​GTT​TCC​AGG​TGG​GC-3′.

Primers for qPCR:

*USP18*-Forward:5′-TGGACAGACCTGCTGCCTTAAC-3′

*USP18*-Reverse:5′-CTGTCCTGCATCTTCTCCAGCA-3′

*IFIT1*-Forward:5′-GCCTTGCTGAAGTGTGGAGGAA-3′

*IFIT1*-Reverse:5′-ATCCAGGCGATAGGCAGAGATC-3′

*ISG15*-Forward:5′-CTCTGAGCATCCTGGTGAGGAA-3′

*ISG15*-Reverse:5′-AAGGTCAGCCAGAACAGGTCGT-3′

*MX1*-Forward:5′-TTAACCTCCACAGAACCGCC-3′

*MX1*-Reverse:5′-GCGGATCAGCTTCTCACCTT-3′

*IFI27*-Forward:5′-CTGGCCAGGATTGCTACAGT-3′

*IFI27*-Reverse:5′-CGGACATCATCTTGGCTGCT-3′

*RSAD2*-Forward:5′-TCCACACAGCCAAAACATCCT-3′

*RSAD2*-Reverse:5′-ACTTGCCCAGGTATTCTCCC-3′

*GAPDH*-Forward:5′-TCGACAGTCAGCCGCATCTTC-3′

*GAPDH*-Reverse:5′-GCGCCCAATACGACCAAATCC-3′.

### Online supplemental material

Fig. S1 provides experimental validation of the identified *NBAS* mutations. Fig. S2 presents additional analysis of the scRNA-seq dataset. Table S1 summarizes the longitudinal clinical course, laboratory findings, interventions, and outcomes of the four patients. Table S2 lists the genetic information of comparator monogenic interferonopathy cases used in Fig. 2 D. Table S3 provides the gene lists used for pathway analyses shown in Fig. 2 E, Fig. 3 E, and Fig. S2 E.

## Supplementary Material

Table S1 shows longitudinal clinical course, laboratory findings, interventions, and outcomes of the four patients.

Table S2 shows variant details of patients with other monogenic interferonopathies.

Table S3 shows gene lists used for pathway analyses shown in Fig. 2 E, Fig. 3 E, and Fig. S2 E.

SourceData F1 is the source file for Fig. 1.

## Data Availability

The *NBAS* variants identified in this study have been deposited in ClinVar under accession numbers VCV000388349.11, VCV004880276.1, VCV004884319.1, VCV004880275.1, and VCV004880277.1. The raw genetic and transcriptional data generated during this study are not publicly available due to ethical and privacy restrictions. The data will be available upon reasonable request. Correspondence and requests for materials should be addressed to the corresponding author.
