# Supplementary material for: Deficiency of NBAS causes immune dysregulation due to hyperactivation of IFN signaling
Source: J Hum Immun. 2026 Oct 28;3(1):e20260018. doi: 10.70962/jhi.20260018 (PMC13715549; doi:10.70962/jhi.20260018)

SourceDataF1C: Co-immunoprecipitation of Flag-tagged wild type/mutated NBAS and Myc-tagged ZW10 that co-expressed in HEK293T cells

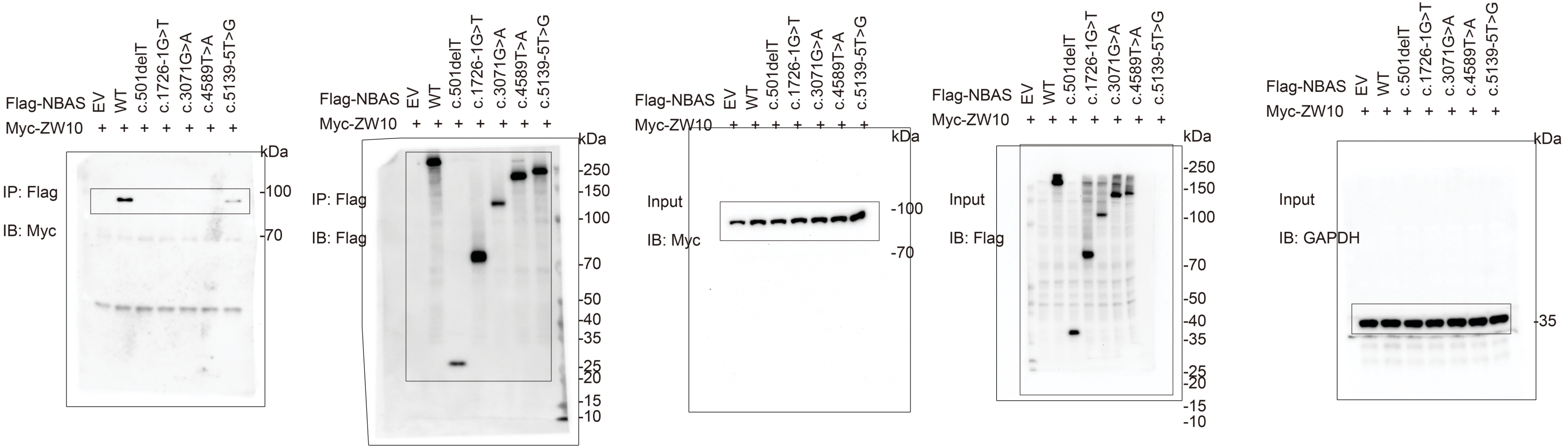

SourceDataF1C: Co-immunoprecipitation of Flag-tagged wild type/mutated NBAS and Myc-tagged RINT1 that co-expressed in HEK293T cells

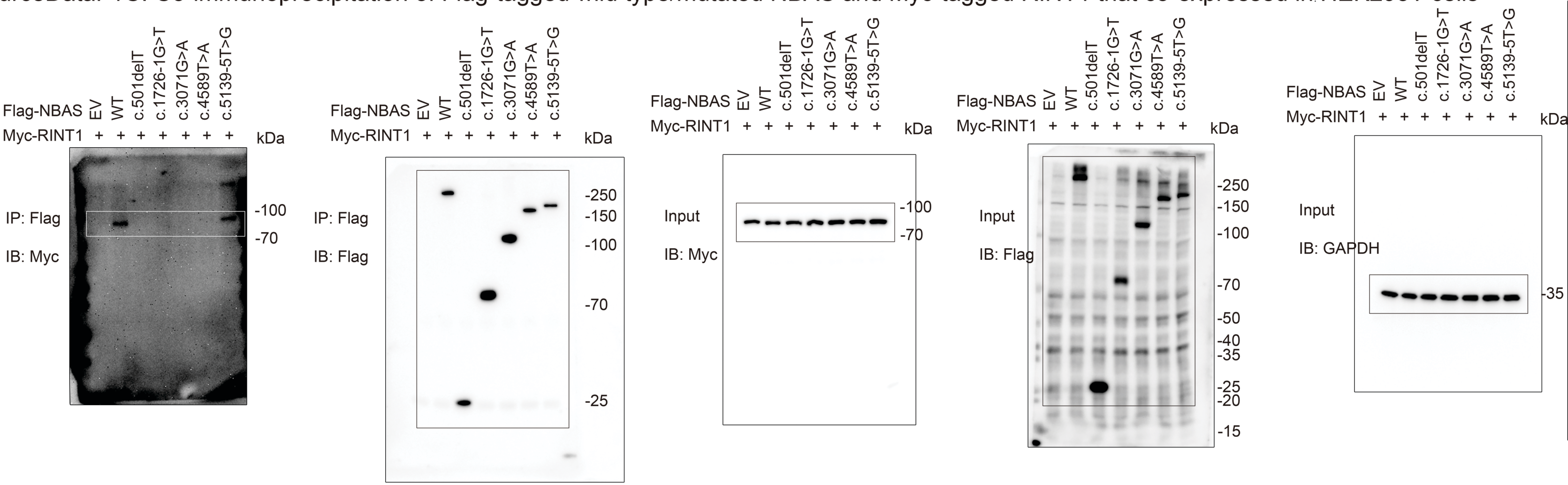

Supplement: Supplementary file 1 [file jhi_20260018_sourcedataf1.pdf]
